# Supplementary material for: Sexual Dysfunction of Patients with Diffuse Low-Grade Glioma: A Qualitative Review of a Neglected Concern
Source: Cancers (Basel). 2022 Jun 20;14(12):3025. doi: 10.3390/cancers14123025 (PMC9221288; doi:10.3390/cancers14123025)
Supplement: Supplementary file 1 [file cancers-14-03025-s001.zip › cancers-1722457-supplementary.pdf]

cancers

MDPI

Supplementary Materials

Sexual Dysfunction of Patients with Diffuse Low-Grade Glioma: A Qualitative Review of a Neglected Concern

Arnaud Lombard [1,2] and Hugues Duffau [3,4,*]

Table S1. Search strategy used in identifying articles from major scientific databases.

Database	Search terms	Items found
PUBMED	(1) "sexual activity" OR "sexual disorder" OR "sexual dysfunction" OR "sexual sphere" OR "sexual health" OR "sexuality"	57016
	(2) "low grade glioma" OR "glioma" OR "LGG" OR "astrocytoma" OR "oligodendroglioma" OR "diffuse glioma" OR "brain tumor"	84401
	(1) And (2)	16
SCOPUS	(1) "sexual activity" OR "sexual disorder" OR "sexual dysfunction" OR "sexual sphere" OR "sexual health" OR "sexuality"	458023
	(2) "low grade glioma" OR "glioma" OR "LGG" OR "astrocytoma" OR "oligodendroglioma" OR "diffuse glioma" OR "brain tumor"	617239
	(1) And (2)	1217
COCHRANE	(1) "sexual activity" OR "sexual disorder" OR "sexual dysfunction" OR "sexual sphere" OR "sexual health" OR "sexuality"	21527
	(2) "low grade glioma" OR "glioma" OR "LGG" OR "astrocytoma" OR "oligodendroglioma" OR "diffuse glioma" OR "brain tumor"	6595
	(1) And (2)	26
Clinicaltrials.gov	("sexual activity" OR "sexual disorder" OR "sexual dysfunction" OR "sexual sphere" OR "sexual health" OR "sexuality") AND ("sexual activity" OR "sexual disorder" OR "sexual dysfunction" OR "sexual sphere" OR "sexual health" OR "sexuality")	10
